# Patients Prefer Human Empathy, but Not Always Human Wording: A Single-Blind Within-Subject Trial of GPT-Generated vs. Clinician Discharge Texts in Emergency Ophthalmology

**DOI:** 10.3390/clinpract15110208

**Published:** 2025-11-14

**Authors:** Dea Samardzic, Jelena Curkovic, Donald Okmazic, Sandro Glumac, Josip Vrdoljak, Marija Skara Kolega, Ante Kreso

**Affiliations:** 1 Department of Ophthalmology, University Hospital of Split, 21000 Split, Croatia; 2 Department of Anesthesiology and Intensive Care, University Hospital of Split, 21000 Split, Croatia; 3 Department for Pathophysiology, University of Split School of Medicine, 21000 Split, Croatia; josip.vrdoljak@mefst.hr; 4 Department of Ophtalmology, Poliklinika Skara Kolega, 21000 Split, Croatia

**Keywords:** ophthalmology, patient education, discharge instructions, large language models, empathy, emergency ophthalmology

## Abstract

**Background/Objectives:** Written discharge explanations are crucial for patient understanding and safety in emergency eye care, yet their tone and clarity vary. Large language models (LLMs, artificial intelligence systems trained to generate human-like text) can produce patient-friendly materials, but direct, blinded comparisons with clinician-written texts remain scarce. This study compared patient perceptions of a routine clinician-written discharge text and a GPT-5-generated explanation, where GPT-5 (OpenAI) is a state-of-the-art LLM, based on the same clinical facts in emergency ophthalmology. The primary objective was empathy; secondary outcomes included clarity, detail, usefulness, trust, satisfaction, and intention to follow advice. **Methods:** We conducted a prospective, single-blind, within-subject study in the Emergency Ophthalmology Unit of the University Hospital Centre Split, Croatia. Adults (*n* = 129) read two standardized texts (clinician-written vs. GPT-5-generated), presented in identical format and in randomized order under masking. Each participant rated both on Likert scales with 1–5 points. Paired comparisons used Wilcoxon signed-rank tests with effect sizes, and secondary outcomes were adjusted using the Benjamini–Hochberg false discovery rate. **Results:** Empathy ratings were lower for the GPT-5-generated text than for the clinician-written text (means 3.97 vs. 4.30; mean difference −0.33; 95% CI −0.44 to −0.22; *p* < 0.001). After correcting for multiple comparisons, no secondary outcome differed significantly between sources. Preferences were evenly split (47.8% preferred GPT-5 among those expressing a preference). **Conclusions:** In emergency ophthalmology, GPT-5-generated explanations approached clinician-written materials on most perceived attributes but were rated less empathic. A structured, human-in-the-loop workflow—in which LLM-generated drafts are reviewed and tailored by clinicians—appears prudent for safe, patient-centered deployment.

## 1. Introduction

Effective discharge communication is central to patient safety, adherence, and satisfaction, yet written materials frequently exceed recommended readability levels and vary in completeness, particularly in time-pressured emergency settings such as ophthalmic urgent care [1]. Recent evaluations show that standard electronic health record (EHR) discharge documents often fall short of health literacy guidance, underscoring the need for formats that are easier to read and act on [1]. Within ophthalmology, where patients commonly face acute anxiety and visual discomfort, improving the clarity and usability of written explanations may be especially valuable for recall and self-management.

Generative large language models (LLMs) have emerged as candidate tools to translate clinical documentation into patient-friendly language. In hospital medicine, LLM-based transformations of discharge summaries increased readability and understandability in controlled evaluations [2], and early trials and pilot studies suggest potential gains in patient comprehension and usability of discharge instructions [3,4]. At the same time, safety concerns are non-trivial: an npj Digital Medicine study identified potentially harmful issues (including hallucinations or incorrect actions) in a meaningful minority of AI-generated discharge instructions, supporting the need for human oversight [5]. Together, these findings suggest promise for LLM-assisted patient education but highlight a critical implementation trade-off between accessibility and factual safety.

Beyond readability, several studies have examined how LLMs compare with clinicians on quality and tone. In a widely cited JAMA Internal Medicine study, licensed clinicians preferred chatbot responses to physicians’ answers to patient questions, rating the chatbot higher for both quality and empathy [6]. Focusing on eye care, a JAMA Network Open study found that LLM-generated advice in response to patient-posed ophthalmology questions was comparable to ophthalmologists’ responses [7], and JAMA Ophthalmology reports show LLMs achieving respectable performance on ophthalmic management questions and multimodal image-based cases, though performance varies by subspecialty and task complexity [8,9]. Collectively, the existing literature indicates that LLMs can produce content that patients and experts sometimes judge favorably, yet none of these studies directly evaluate patient-facing written explanations against clinician-written documents for the same individual encounter under blinded conditions.

Despite growing evidence that LLMs can generate clinically relevant ophthalmology content and achieve performance comparable to ophthalmologists on specific diagnostic or question-answering tasks, prior research has primarily focused on expert evaluations or comprehension metrics rather than patient perceptions. To our knowledge, no study has directly compared patient-facing written explanations produced by clinicians and by LLMs for the same clinical cases under blinded conditions. Addressing this gap, we designed a prospective, single-blind, within-subject comparison to assess how patients perceive empathy, clarity, and usefulness of clinician-written versus GPT-5-generated discharge texts in emergency ophthalmology.

Empathy was chosen as the primary outcome because it plays a pivotal role in patient comprehension, trust, and adherence to discharge instructions, particularly in emergency ophthalmology, where patients often experience acute anxiety and uncertainty. Written communication that conveys empathy can improve perceived support, reduce distress, and enhance the likelihood of patients following therapeutic and safety advice.

Therefore, the present study addresses this gap by performing a single-blind, within-subject comparison of a routine clinician-written discharge text versus a GPT-generated written explanation derived from the same clinical facts in an ophthalmic emergency setting. We focus on patient-reported empathy (primary outcome), and clarity, detail, usefulness, trust, satisfaction, and adherence intention (secondary outcomes). By standardizing formatting, randomizing text order, and masking sources, we aim to isolate content effects while acknowledging prior evidence of both benefits (readability and perceived empathy) and risks (possible safety errors). Our hypothesis is that GPT-generated explanations will be non-inferior to clinician-written texts regarding empathy and may improve perceived clarity and usefulness, while any safety-relevant discrepancies will be identifiable via structured oversight.

## 2. Materials and Methods

### 2.1. Study Design and Setting

We conducted a prospective, single-blind, within-subject study in the Emergency Ophthalmology Unit of the University Hospital Centre Split (KBC Split), Croatia. The objective was to compare patients’ perceptions of a clinician-written discharge text and a GPT-generated written explanation based on the same clinical facts for the index visit. The Ethics Committee of the University Hospital of Split approved the protocol (Class: 520-03/25-01/221 Reg. No.: 2181-147/01-06/LJ.Z.-25-02).

The study adhered to the Declaration of Helsinki. Participation was voluntary and anonymous; completion of the questionnaire constituted informed consent.

### 2.2. Participants

Eligible participants were adults (≥18 years) who completed their clinical assessment and were stable for discharge. Exclusion criteria were time-critical emergencies requiring immediate intervention, cognitive or visual limitations preventing meaningful reading of written materials, insufficient proficiency in Croatian for written comprehension, and minors.

Included cases were consecutive adult patients presenting with anterior segment inflammatory, infectious, or minor traumatic conditions suitable for same-day discharge, such as conjunctivitis, corneal foreign body, superficial keratitis, hordeolum/chalazion, anterior uveitis, and minor ocular trauma. Exclusion criteria were sight-threatening emergencies, need for admission, or severe trauma. The full distribution of diagnoses is provided in the Supplementary Materials (Table S1). Participants were recruited consecutively between 29 August and 19 September 2025, during investigator duty shifts in the Emergency Ophthalmology Unit, which included both weekdays and weekends, typically during daytime and early evening hours, but not overnight.

### 2.3. Stimuli (Text Sources)

Clinician-written text: The treating ophthalmologist prepared the routine written conclusion/discharge note as per standard practice (no content changes were introduced for the study).

GPT-generated text: Using the same clinical facts (diagnosis, therapy with doses/frequency/duration, follow-up timing, red flag symptoms with actions, and expectations), the researcher generated a patient-facing explanation with GPT-5 using a fixed system prompt. Target length was 130–150 words (~140 words) at B1 readability in Croatian.

### 2.4. Prompt and Input to GPT-5

The generation process used a fixed system prompt (Croatian, provided verbatim in the Supplementary Materials Box S1) and PHI-free continuous USER input extracted from the clinician’s record. All outputs were generated using GPT-5 (OpenAI, API release May 2025) via the chat completion interface, with default decoding parameters (temperature = 1.0; top-*p* = 1.0). A single fixed system prompt was used verbatim for all generations without any iterative prompt tuning or parameter modification. An illustrative example of a paired clinician-written and GPT-5-generated text (original Croatian and English translation) is provided in the Supplementary Materials (Example S1). The prompt specified a target readability of B1 level, corresponding to the ‘intermediate’ proficiency level under the Common European Framework of Reference for Languages [10]. B1 readability was selected as it aligns with recommended health literacy targets for patient education materials in Europe, balancing clarity and informational completeness.

#### 2.4.1. Box 1: English Translation of the Prompt (For Reporting Only)

You are a patient educator in ophthalmology. Write in Croatian, B1 readability, warm and clear, professional. Length 130–150 words (target 140). Do not introduce diagnoses/tests/therapies not present in the input. Never contradict the clinician’s note. Do not mention authorship or that you are AI; no disclaimers; avoid ‘we/our team’. Write doses with units (e.g., ‘1 drop 3× daily for 7 days’).

The input will be a free, continuous text (record/visit summary) containing: diagnosis, prescribed therapy (drug, dose, frequency, duration), follow-up timing, red-flag symptoms with actions, expected course, and any special instructions. Use only what is explicitly provided. If an element is missing, omit that subsection (do not invent content).

Follow exactly this output FORMAT and nothing else:

Diagnosis: (1–2 short lay sentences)

What it is/is not:

- (point 1)
- (point 2)
- (point 3)

Therapy:

- (drug 1—dose, frequency, duration; key instructions)
- (drug 2—dose, frequency, duration; key instructions)

Red flags:

- (symptom 1 and what to do)
- (symptom 2 and what to do)

Follow-up and expectations: (1–2 sentences)

End without a closing remark, signature, or links.

#### 2.4.2. USER Input (Source of Content)

The USER input to GPT-5 was a continuous excerpt from the clinician’s record (Croatian), containing only the clinical facts listed above. All personally identifiable information (PHI), such as name, ID, address, and date of birth, was removed before submission. No PHI was entered into GPT-5 at any time. The input to GPT-5 consisted of a concise, de-identified summary extracted from the clinician’s discharge note, limited to diagnosis, therapy (drug name, dose, frequency, and duration), follow-up timing, red flag symptoms with corresponding actions, and expected course. The full admission record or detailed clinical history was not used.

### 2.5. Standardization of Presentation and Blinding

To preserve blinding and isolate content effects, both texts were formatted in an identical template (same font, size, margins, alignment, spacing, and section headings), with no logos, stamps, signatures, or headers. Length harmonization targeted 130–150 words for both texts when feasible; for the clinician-written text, only micro-edits of layout (e.g., sentence splits or bulleting) were permitted without altering meaning. Any source-revealing phrases (e.g., “as your doctor…” or “as an AI…”) were replaced with neutral phrasing when present. Texts were labeled “TEXT A” and “TEXT B”. Patients always read A and then B. A 1:1 randomization determined whether Text A was the clinician-written version or the GPT-5-generated version. Administering staff were not the treating clinicians. Analysis datasets used masked variables (source_A: 1 = clinician; 2 = GPT), with the randomization key stored separately. Although clinicians initially wrote their routine discharge text in free-text format, for the experiment, both the clinician-written and GPT-5-generated texts were reformatted into the same structured template (section headings and bullet points as needed), with only micro-edits of layout and no changes to clinical content.

### 2.6. Procedure

(1) The clinician completed the routine clinical assessment and wrote the standard discharge text.
(2) The researcher prepared a PHI-free continuous input and generated text with the fixed prompt.
(3) Both texts were standardized into the common template and produced as TEXT A and TEXT B per randomization.
(4) Participants read TEXT A and then TEXT B and immediately completed the questionnaire (see Section 2.7).
(5) After completion, participants were offered a brief debrief stating that one of the texts was AI-generated.

### 2.7. Outcomes and Instrument

Participants rated each text using a 10-item Likert instrument (1 = strongly disagree; 5 = strongly agree). The 10-item patient questionnaire was developed specifically for this study, with items conceptually derived from prior empathy and communication quality frameworks but adapted for written discharge materials. Content validity was confirmed through expert review by three experienced clinicians, and internal consistency in the present sample was high (Cronbach’s α = 0.89). The primary construct was empathy (3 items: empathic tone, feeling understood, and addressed concerns). The 3-item empathy composite was developed specifically for this study to reflect perceived empathy in written discharge explanations, covering empathic tone, feeling understood, and addressing patient concerns. Item wording was informed by domains from established empathy frameworks but adapted for written text evaluation. Secondary constructs included detail (1 item), clarity (1), usefulness (2: therapy instructions and when to seek care), trust (1), adherence intention (1), and overall satisfaction (1). After both blocks, participants indicated an overall preference (TEXT A, TEXT B, or no preference). Blinding was checked by asking “Who do you think wrote TEXT A? (Clinician/GPT/Don’t know)” and a confidence rating (1–5). Covariates included age, sex, education, urbanicity, native language (Croatian, yes/no), reading frequency, font comfort, digital literacy (1–5), and prior AI use (1–5).

### 2.8. Randomization and Allocation Concealment

A computer-generated list with variable block sizes provided a 1:1 allocation of A = clinician versus A = GPT. Allocation was concealed using sequentially numbered, opaque, sealed envelopes or pre-assigned tablet codes. The randomization log (A/B → source) was stored offline and accessible only to the data manager.

### 2.9. Sample Size and Power

We powered the study for a small-to-moderate within-subject effect on the primary outcome (Cohen’s *d_z_* ≈ 0.25–0.30; two-sided α = 0.05; 1-β = 0.80), yielding a target of *N* ≈ 90–130 analyzable participants. To account for attrition and exclusions, we planned to enroll 130–200 participants.

### 2.10. Data Management, Privacy, and Security

Data were pseudonymized on entry; survey records contained no direct identifiers. USER inputs to GPT-5 excluded PHI by design. Analysis datasets used only A/B labels and covariates; the randomization key was stored separately. Paper forms (if any) were stored in locked cabinets; electronic data were stored on secure institutional servers with role-based access.

### 2.11. Statistical Analysis

We conducted a complete-case analysis: only questionnaires with 100% item completion for all outcomes and covariates were included in the analyses; no imputation or prorating was performed.

For the primary outcome (empathy; GPT-5 vs. clinician), normality of paired differences was assessed using the Shapiro–Wilk test. If normality held, the comparison used a paired *t*-test, with effects reported as mean difference (GPT-5–clinician), 95% confidence interval (CI), and Cohen’s *d_x_*. If the normality assumption was violated (as in the observed data), the Wilcoxon signed-rank test was used instead, with effects expressed as mean difference with bootstrap 95% CI and matched rank-biserial correlation (*r*) as the effect size.

Secondary outcomes were analyzed with parallel paired tests (*t*-test or Wilcoxon, as appropriate) and adjusted for multiple comparisons using the Benjamini–Hochberg false discovery rate (FDR) across secondary endpoints.

Text source preference (GPT-5 vs. clinician vs. no preference) was analyzed using multinomial logistic regression, with optional adjustment for covariates.

Exploratory heterogeneity analyses assessed associations between the within-participant paired differences (Δ = GPT-5 − clinician) and participant characteristics (age, sex, education, residence, digital literacy, and AI tool experience) using Spearman’s ρ (with bootstrap 95% CIs and BH–FDR control within each outcome). Differences by sex were tested using the Mann–Whitney test with rank-biserial *r*, and differences by residence were determined using the Kruskal–Wallis test with the epsilon-squared value (ε^2^) as the effect size. As a robustness check, linear mixed-effects or paired regression models were fitted including these covariates and their interactions with the source (GPT-5 vs. clinician).

Internal consistency for multi-item scales was evaluated using Cronbach’s α and McDonald’s ω where applicable.

All analyses were conducted in R (version 4.3.2; R Core Team, Vienna, Austria).

## 3. Results

### 3.1. Participants

A total of 129 respondents were analyzed (age: 49.73 (17.72) years; 55.8% male). Digital literacy (1–5): median [IQR] = 4 [2]; AI tool experience (1–5): median [IQR] = 3 [2]. Detailed categorical distributions for sex, education, residence, reading frequency, difficulty reading small text, and preferred information format are presented in Table 1.

### 3.2. Primary Outcome: Empathy

The mean empathy score (3-item composite; 1–5) was lower for the GPT-5-generated text than for the clinician-written text (3.97 ± 0.75 vs. 4.30 ± 0.68), with a mean difference of −0.33 (95% CI −0.44 to −0.22). The distribution of paired differences deviated from normality; therefore, the Wilcoxon signed-rank test was used (W = 1198, *p* < 0.001). The matched rank-biserial correlation was *r* = −0.35, indicating a medium effect favoring the clinician-written text. By convention, negative values of r(rb) indicate higher ratings for clinician-written texts, and positive values favor GPT-5-generated texts. Internal consistency of the empathy subscale was acceptable (Cronbach’s α = 0.78 for GPT-5-generated text and 0.74 for clinician-written text).

In a linear mixed-effects model with a random intercept for participants, the source effect (GPT-5 vs. clinician) remained statistically significant (β = −0.33, 95% CI [−0.44, −0.22], *p* < 0.001). No significant interactions were observed between source and age, education, sex, digital literacy, or AI tool experience (all *p* > 0.10), indicating that the observed empathy difference was consistent across participant subgroups (Table S1).

A forest plot of bootstrap confidence intervals for correlations between Δ empathy and participant characteristics is presented in Figure S1. This visualization confirms that all 95% CIs for Spearman’s ρ crossed zero except for age (*ρ* = 0.21), supporting the conclusion of minimal heterogeneity in empathy differences.

### 3.3. Secondary Outcomes

After controlling for multiple comparisons via the Benjamini–Hochberg FDR procedure, no secondary outcome reached statistical significance. Individual paired comparisons are summarized in Table 2.

### 3.4. Text-Source Preferences

**Figure 1:**
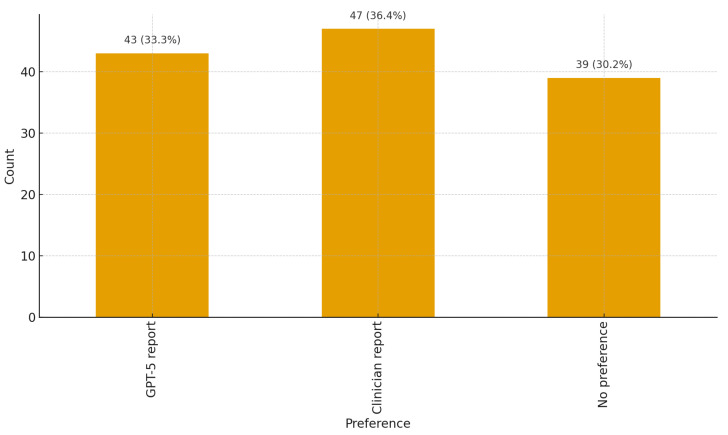
Distribution of preferences for GPT-5-generated versus clinician-written text. Bars show percentages of participants preferring each source or reporting no preference.

In the full sample, 36.4% (*n* = 47) preferred the clinician text, 33.3% (*n* = 43) preferred the GPT-5-generated text, and 30.2% (*n* = 39) reported no preference. Among respondents who stated a preference (*N* = 90), 47.8% (43/90) preferred the GPT-5-generated text; the two-sided binomial test (*p* = 0.752) indicates no difference in preference between the GPT-5-generated and clinician-written text. Visualization is provided in Figure 1.

### 3.5. Subgroup Analyses and Covariate Associations

We examined associations between the within-participant paired differences (Δ = GPT-5 − clinician) and age, education, digital literacy, and AI tool experience using Spearman’s ρ with bootstrap 95% CIs and Benjamini–Hochberg false discovery rate (FDR) control within outcome; sex and residence were tested using Mann–Whitney (rank-biserial r) and Kruskal–Wallis (ε^2^) tests, respectively. For the primary outcome (empathy), older age showed a modest positive association with Δ (ρ = 0.21, 95% CI [0.06, 0.37], *p* = 0.016; FDR *p* = 0.063), indicating a smaller empathy deficit for GPT-5 among older participants. All other covariate associations for empathy were non-significant after FDR adjustment. Across secondary outcomes, no associations survived FDR; sex and residence showed no detectable differences in Δ (all *p* > 0.25 and ε^2^ ≈ 0). These findings suggest limited heterogeneity of the GPT-5 vs. clinician contrast across measured participant characteristics. Results were consistent when using an adjusted mixed/fixed-effects model (Table S1).

## 4. Discussion

In this single-blind, within-subject study in emergency ophthalmology, patients perceived the clinician-written text as more empathic than the GPT-generated explanation, whereas clarity, detail, usefulness, trust, satisfaction, and intention to follow advice were broadly similar between sources. Among participants who expressed a preference, choices were roughly evenly split. Heterogeneity was limited: older patients tended to perceive a smaller empathy gap; education, sex, place of residence, digital literacy, and prior AI experience did not meaningfully change the pattern.

Previous studies comparing clinician-written and LLM-generated responses have generally assessed unconstrained Q&A exchanges rather than patient-specific discharge explanations. Some have found higher perceived empathy for AI-generated replies in online or oncology settings [6,11]. These contexts differ substantially from the blinded, encounter-specific design of the present study. Within ophthalmology, expert evaluations have shown that LLM responses can approximate clinician quality for patient questions but still exhibit generic phrasing or occasional factual errors, underscoring the importance of careful use-case selection and human review [7,9,12].

Although the empathy difference between clinician-written and GPT-5-generated texts reached statistical significance, its clinical magnitude was modest (mean difference of −0.33 on a 5-point scale). This corresponds to roughly one-third of a scale point—detectable by patients but unlikely to alter comprehension, trust, or adherence in isolation. However, even small reductions in perceived empathy can influence engagement and satisfaction, particularly in emotionally charged emergency settings. From a clinical perspective, these findings support a human-in-the-loop approach in which LLM-generated discharge materials are reviewed and personalized by clinicians to preserve empathetic tone while benefiting from automation.

The internal consistency of the patient questionnaire was high (Cronbach’s α = 0.89), supporting the reliability of the measured perceptions. Given the within-subject design, where each participant rated both text versions, the results can be considered reproducible at the group level, though individual perceptions may vary between encounters.

This study extends prior LLM–clinician comparisons by focusing on real emergency ophthalmology encounters and direct patient evaluations of discharge explanations rather than expert or simulated assessments. To our knowledge, it is the first prospective, blinded, within-subject design in which patients compared clinician-written and GPT-5-generated explanations based on identical clinical facts. These findings contribute to understanding how large language models may support—but not replace—clinician communication in acute care, where clarity and empathy must coexist under time pressure.

Beyond perceived quality, the readability and usability of discharge materials are critical. A JAMA Network Open study showed that transforming inpatient discharge summaries with an LLM substantially improved readability and understandability while cautioning that accuracy, completeness, and safety require clinician review, consistent with a human-in-the-loop approach [2]. Complementing this, an npj Digital Medicine analysis flagged potentially harmful issues in a notable fraction of AI-generated discharge instructions, reinforcing the need for guardrails and oversight [5]. In addition, perceptions of credibility and acceptance can be influenced by whether readers believe a text was authored by AI, highlighting framing and disclosure effects to consider in deployment [13].

Our findings also sit alongside emerging evidence from the same clinical context showing that state-of-the-art LLMs can generate diagnoses and treatment plans with accuracy comparable to ophthalmologists on real emergency cases, supporting their role as decision-support rather than autonomous systems. This convergence, technical adequacy alongside a residual empathy gap, argues for workflows where clinicians retain final oversight while leveraging LLMs for structure and efficiency [14].

For emergency ophthalmology, templated GPT-generated explanations can approach clinician materials on clarity and perceived usefulness but may lack the relational tone patients value. Practical implementation should therefore pair auto-drafting with mandatory clinician edits to verify content; infuse empathic, patient-centered language; and tailor the text to the individual’s concerns. Health systems adopting these tools should incorporate safety guardrails (e.g., medication and dosing checks; curated ‘red flag’ libraries; and prompts that suppress speculation) and post-deployment auditing for rare but consequential errors [2].

The small age-related moderation of empathy (older patients perceiving a smaller GPT deficit) may reflect differences in expectations for tone or reading strategies. Notably, digital and AI literacy did not materially shift perceptions, suggesting that the empathy gap is not simply a function of users’ technical familiarity.

Strengths include the within-subject, single-blind design, standardized formatting and readability level, prospectively defined outcomes with acceptable reliability, and convergent results across nonparametric paired tests and a replication mixed-effects model. Limitations include a single-center setting, Croatian-language materials, and a focus on short-term perceptions rather than measured comprehension, behavior, or clinical outcomes. Although sources were masked, subtle linguistic cues may still have hinted at authorship. While texts were constrained to recorded facts, we did not adjudicate factual accuracy as a primary endpoint in this experiment. The present study applied a structured, templated format to both clinician-written and GPT-5-generated texts for consistency and safety; future research will compare unconstrained free-text outputs from clinicians and large language models to disentangle how structure and narrative flow contribute to perceived empathy.

Multicenter, multilingual trials should test hybrid workflows (LLM draft and clinician edit) on objective comprehension, medication adherence, unplanned revisits, and safety outcomes, alongside time-and-cost metrics. Technique-oriented work should evaluate style transfer tuned to clinical empathy and domain-specific guardrails and continue benchmarking LLMs on ophthalmology-specific tasks—including those requiring multimodal input—under rigorous human oversight [9]. Future studies should also test whether disclosure versus non-disclosure of AI authorship affects patient trust and uptake [13] and expand benchmarking beyond text-only tasks to complex ophthalmology scenarios reported to induce generic phrasing or errors [12,15].

## 5. Conclusions

Clinician-written discharge text was perceived as more empathic than GPT-generated text, while other attributes were largely similar. Preferences were roughly balanced, and subgroup effects were minimal. These results support a human-in-the-loop workflow: LLMs draft, while clinicians review for accuracy, safety, and tone. Future multicenter studies should test whether this hybrid approach improves comprehension, adherence, and safety and assess how disclosure of AI authorship affects trust. Key limitations include the single-center design, Croatian-language materials, and focus on short-term perceptions rather than clinical outcomes, which future multicenter, multilingual trials should address.

## Figures and Tables

**Table 1:** Categorical distributions (*n* = 129).

Category	Subcategory	*N* (%)
Sex	Female	57 (44.2)
	Male	72 (55.8)
	Total	129 (100.0)
Education	Primary	5 (3.9)
	Secondary	65 (50.4)
	College/Undergrad	23 (17.8)
	Graduate	32 (24.8)
	Postgraduate	4 (3.1)
	Total	129 (100.0)
Residence	City	99 (76.7)
	Suburban	15 (11.6)
	Rural	11 (8.5)
	Island	4 (3.1)
	Total	129 (100.0)
Reading long texts	Rarely	7 (5.4)
	Sometimes	40 (31.0)
	Often	50 (38.8)
	Daily	32 (24.8)
	Total	129 (100.0)
Difficulty reading small text	Rarely	7 (5.4)
	Sometimes	40 (31.0)
	Often	50 (38.8)
Preferred information format	Verbal	27 (20.9)
	Written	47 (36.4)
	Video	1 (0.8)
	Combination	54 (41.9)
	Total	129 (100.0)

**Table 2:** Paired comparisons of GPT-5-generated vs. clinician-written text (*n* = 129; higher is better; 1–5 scale).

Outcome	GPT-5 (M ± SD)	Clinician (M ± SD)	Δ (GPT-5 − CLIN)	Effect Size r(rb)	*p* Value
Empathy (3 items) ^a^	3.97 ± 0.75	4.30 ± 0.68	−0.33	−0.35	<0.001
Detail (1 item)	4.43 ± 0.63	4.36 ± 0.64	+0.06	+0.08	0.427
Clarity (1 item)	4.40 ± 0.67	4.40 ± 0.67	0.00	0.00	0.946
Usefulness (2 items) ^b^	4.55 ± 0.64	4.60 ± 0.63	−0.04	−0.05	0.486
Trust (1 item)	4.61 ± 0.59	4.52 ± 0.63	+0.09	+0.10	0.267
Adherence intention (1 item)	4.59 ± 0.60	4.60 ± 0.59	−0.01	−0.02	0.827
Satisfaction (1 item)	4.32 ± 0.70	4.44 ± 0.68	−0.12	−0.09	0.251

^a^ Empathy (3 items) is a composite (mean) of three Likert items assessing empathic tone, feeling understood while reading, and whether the text addressed the participant’s concerns. ^b^ Usefulness (2 items) is a composite (mean) of two Likert items capturing applied usefulness: knowing how to apply the therapy and knowing when to seek help. All tests are two-sided Wilcoxon signed-rank tests; effect sizes are matched rank-biserial correlations r(rb). Negative values favor the clinician-written text.

## Data Availability

The original contributions presented in this study are included in the article/Supplementary Materials. Further inquiries can be directed to the corresponding author.

## Supplementary Materials

The following supporting information can be downloaded at https://www.mdpi.com/article/10.3390/clinpract15110208/s1, Box S1. Fixed System Prompt (original, Croatian); Example S1. Example of summary text; Figure S1. Bootstrap 95% confidence intervals for correlations between Δ empathy (GPT-5 − Clinician) and participant characteristics; Table S1. Mixed-effects model for empathy (Δ = GPT-5 − Clinician); Table S2. Mixed-effects model for empathy (Δ = GPT-5 − Clinician).

## Abbreviations

The following abbreviations are used in this manuscript.

AI	Artificial Intelligence
CI	Confidence Interval
FDR	False Discovery Rate
GPT-5	Generative Pre-Trained Transformer, version 5
HITL	Human-in-the-Loop
IQR	Interquartile Range
LLM	Large Language Model
Q&A	Question and Answer
SD	Standard Deviation
