# Supplementary material for: Patients Prefer Human Empathy, but Not Always Human Wording: A Single-Blind Within-Subject Trial of GPT-Generated vs. Clinician Discharge Texts in Emergency Ophthalmology
Source: Clin Pract. 2025 Nov 14;15(11):208. doi: 10.3390/clinpract15110208 (PMC12651557; doi:10.3390/clinpract15110208)
Supplement: Supplementary file 1 [file clinpract-15-00208-s001.zip › Samardzic_et_al_ Example S1.pdf]

Example S1. Example of summary text

Croatian Version

GPT-5–GENERATED TEXT

Dijagnoza: U desnom oku pronađeno je i uklonjeno strano tijelo iz rožnice. Na mjestu ozljede vidljive su površinske promjene, ali oko nije probijeno.

Što jest / što nije:
[illegible]trano tijelo je uspješno uklonjeno
Prisutno je površinsko oštećenje rožnice
Dublji slojevi oka nisu zahvaćeni

Terapija:
Tobrex kapi - 1 kap 5× dnevno kroz 7 dana, zatim prestati
Kloramfenikol mast - 5× dnevno kroz 7 dana, zatim prestati
Okluzija oka - držati zatvoreno zavojem 2 dana

Crvene zastavice:
Naglo pogoršanje vida - odmah potražiti hitnu pomoć
Povećana bol ili obilno suzenje - javiti se na hitni prijem

Kontrola i očekivanja: Kontrola je planirana za 2 dana. Očekuje se postupno cijeljenje rožnice uz pravilnu primjenu terapije.

CLINICIAN-WRITTEN TEXT

Dijagnoza: Pregledani ste zbog stranog tijela rožnice desnog oka.

Što jest / što nije:
To je površinsko oštećenje rožnice.
U pravilu zacjeljuje bez trajnih oštećenja za vid.

Terapija:
Ambulantno je odstranjeno strano tijelo te su ordinirane antibiotske kapi.
Potrebno je stavljati terapiju: Tobrex kapi 5 puta dnevno po jednu kap te nakon par minuta staviti Kloramfenikol mast oko 0,5 cm sa unutarnje strane donje vjeđe.
Planirano je trajanje terapije tijekom 7 dana.
Nakon što stavite terapiju savjetuje se držati oko zatvoreno sljedeća dva dana - svaki put stavite čisti prevoj.

Crvene zastavice: U slučaju ispada vidne oštrine, izražene bolnosti ili pojave znakova upale - javite se na hitni prijem!

Kontrola i očekivanja: Kontrolni pregled je planiran za 2 dana. Očekuje se potpuni oporavak nakon korištenja terapije tijekom 7 dana.

English version

GPT-5–GENERATED TEXT

Diagnosis: A foreign body was found and removed from the cornea of the right eye. At the site of injury, superficial changes are visible, but the eye was not penetrated.

What it is / what it is not:
The foreign body has been successfully removed
There is a superficial corneal injury
The deeper layers of the eye are not affected
Therapy:
Tobrex drops - 1 drop 5× daily for 7 days, then discontinue
Chloramphenicol ointment — 5× daily for 7 days, then discontinue
Eye occlusion - keep the eye closed with a bandage for 2 days

Red flags:
�udden deterioration of vision - seek emergency medical help immediately
Increased pain or excessive tearing - report to the emergency department

Follow-up and expectations: Follow-up is scheduled in 2 days. Gradual corneal healing is expected with proper use of therapy.

CLINICIAN-WRITTEN TEXT

Diagnosis: You were examined due to a foreign body in the cornea of the right eye.

What it is / what it is not:
This is a superficial corneal injury.
It usually heals without permanent vision damage.

Therapy:
The foreign body was removed on an outpatient basis, and antibiotic drops were prescribed.
Therapy instructions: apply *Tobrex* drops 5 times a day, one drop each time, and after a few minutes apply *Chloramphenicol* ointment (approximately 0.5 cm) to the inner side of the lower eyelid.
The planned duration of therapy is 7 days.
After applying the medication, it is advised to keep the eye closed for the next two days - each time use a clean dressing.

Red flags: In case of loss of visual acuity, significant pain, or signs of inflammation - report to the emergency department!

Follow-up and expectations: A follow-up examination is scheduled in 2 days. Full recovery is expected after completing the 7-day therapy.
