# Supplementary material for: Patients Prefer Human Empathy, but Not Always Human Wording: A Single-Blind Within-Subject Trial of GPT-Generated vs. Clinician Discharge Texts in Emergency Ophthalmology
Source: Clin Pract. 2025 Nov 14;15(11):208. doi: 10.3390/clinpract15110208 (PMC12651557; doi:10.3390/clinpract15110208)
Supplement: Supplementary file 1 [file clinpract-15-00208-s001.zip › Samardzic_et_al_Table S1.pdf]

Supplementary Table

Table S1. Distribution of ophthalmic diagnoses among patients included in the study (n = 129).

Diagnosis	n (%)
Conjunctivitis (bacterial, viral, allergic)	34 (26.4%)
Corneal foreign body / superficial keratitis	28 (21.7%)
Hordeolum / chalazion / blepharitis	18 (14.0%)
Subconjunctival hemorrhage	11 (8.5%)
Anterior uveitis / iritis	10 (7.8%)
Corneal abrasion (non–foreign body)	8 (6.2%)
Minor blunt ocular trauma	7 (5.4%)
Dry eye exacerbation	3 (2.3%)
Other acute conditions [a] (chemical irritation, contact-lens related, retinal rupture, etc.)	20 (15.5%)
**Total**	**129 (100%)**

Legend

[a] *Other* category includes all diagnoses that occurred fewer than three times, such as chemical irritation, contact-lens related inflammation, and retinal rupture.
