# Supplementary material for: Patients Prefer Human Empathy, but Not Always Human Wording: A Single-Blind Within-Subject Trial of GPT-Generated vs. Clinician Discharge Texts in Emergency Ophthalmology
Source: Clin Pract. 2025 Nov 14;15(11):208. doi: 10.3390/clinpract15110208 (PMC12651557; doi:10.3390/clinpract15110208)
Supplement: Supplementary file 1 [file clinpract-15-00208-s001.zip › Samardzic_et_al_Table S2.pdf]

Supplementary Table S2. Mixed-effects model for empathy (Δ = GPT-5 − Clinician).

**Predictor**	**β**	**95% CI**	**p value**
Source (GPT-5 vs Clinician)	−0.331	[−0.440, −0.222]	<0.001
Age (years)	+0.001	[−0.002, +0.004]	0.489
Sex (M=1, F=2)	−0.021	[−0.134, +0.093]	0.720
Education (1–5)	−0.019	[−0.053, +0.016]	0.284
Digital literacy (1–5)	−0.031	[−0.072, +0.010]	0.139
AI tool experience (1–5)	−0.011	[−0.047, +0.024]	0.532
Source × Digital literacy	+0.014	[−0.020, +0.049]	0.407
Source × AI experience	+0.008	[−0.027, +0.042]	0.661

Model: mixed-effects regression with participant as random intercept; dependent variable = empathy score (1–5). CI = 95% bootstrap confidence interval.
