# Supplementary figures and images for: Patients Prefer Human Empathy, but Not Always Human Wording: A Single-Blind Within-Subject Trial of GPT-Generated vs. Clinician Discharge Texts in Emergency Ophthalmology
Source: Clin Pract. 2025 Nov 14;15(11):208. doi: 10.3390/clinpract15110208 (PMC12651557; doi:10.3390/clinpract15110208)

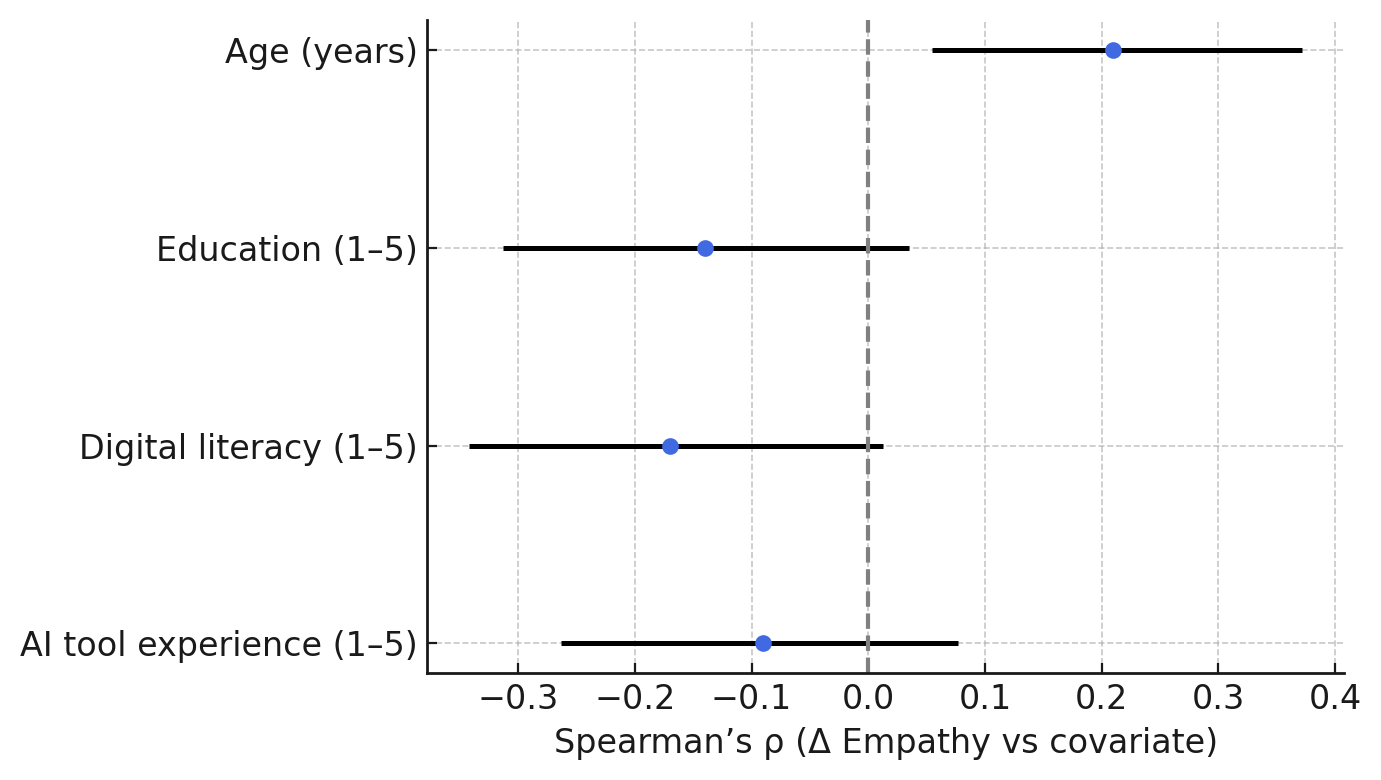

Supplement: Supplementary file 1 [file clinpract-15-00208-s001.zip › Samardzic_et_al_Figure S1.png]
